# “I can do math!”: A self‐regulated learning intervention to enhance math‐related motivational factors and performance in middle school

**DOI:** 10.1111/bjep.70034

**Published:** 2025-09-18

**Authors:** Federica Granello, Alessandro Cuder, Eleonora Doz, Sandra Pellizzoni, Maria Chiara Passolunghi

**Affiliations:** ^1^ Department of Life Sciences University of Trieste Trieste Italy

**Keywords:** intervention, math motivation, math performance, middle school, self‐regulated learning

## Abstract

**Introduction:**

Self‐regulated learning (SRL) interventions have been widely recognized for their potential to enhance students' academic achievement; however, their effects on math‐related motivational constructs remain less explored. This study investigated the impact of an SRL intervention on multiple math‐related motivational factors (math perseverance, math self‐efficacy, math utility value, STEM vocational interest and theory of intelligence) and math performance among Year 6 and Year 7 middle school students (*N* = 382), assessing outcomes both immediately after the intervention (post‐test) and 6 months later (follow‐up).

**Methods:**

Students in the SRL intervention group engaged in six sessions focusing on planning, performance monitoring and self‐evaluation strategies. In contrast, a control group practiced the same math exercises without explicit SRL training.

**Results:**

Findings revealed that, at post‐test, the SRL group showed improvements in math perseverance, math utility value, and theory of intelligence, as well as in math performance, compared to the control group. No differences were observed in math self‐efficacy and STEM vocational interest. However, gains in math perseverance, theory of intelligence and math performance persisted at the 6‐month follow‐up, suggesting persistent benefits of the SRL intervention.

**Conclusion:**

These findings underscore the value of integrating SRL interventions into regular math instruction and highlight the potential of such interventions to foster both math motivation and performance.

## INTRODUCTION

In modern societies, mathematical competence is increasingly essential, given its strong association with favourable educational and career outcomes (Bynner & Parsons, 1997; Rivera‐Batiz, 1992), economic stability (Gerardi et al., 2013; Gross et al., 2009), and physical and mental well‐being (Furlong et al., 2016; Gross et al., 2009). While high‐quality instruction and basic skill proficiency contribute to success in secondary‐level mathematics (Cleary & Kitsantas, 2017), a growing body of research emphasizes the crucial role of motivational factors in sustaining academic engagement and performance in mathematics (Burnette et al., 2013; Credé et al., 2017; Cuder et al., 2024; Lauermann et al., 2017; Marsh et al., 2005; Renninger & Hidi, 2016). Therefore, there is a need for interventions capable of promoting both math‐related motivational factors and math performance (Granello et al., 2025; Zakariya, 2022). In this regard, self‐regulated learning (SRL), defined as the capacity to develop self‐directed thoughts and behaviours that are deliberately initiated and adjusted to achieve personal goals (Pintrich et al., 2000; Sinkkonen & Tapani, 2024; Zimmerman, 1989), has emerged as a central process in learning. Empirical findings suggest that SRL fosters higher goal setting, helps students overcome academic challenges and encourages mastery over performance goals, thus supporting motivation in mathematics (Burnette et al., 2013; Diseth, 2011; Ford et al., 1998; Howell & Watson, 2007; Lourenço & Paiva, 2024; McWhaw & Abrami, 2001; Somuncuoglu & Yildirim, 1999; Wang et al., 2025).

This study aimed to evaluate the effectiveness of an SRL intervention in enhancing math‐related motivational factors (i.e., math perseverance, math self‐efficacy, math utility value, STEM vocational interest and theory of intelligence) and math performance among middle school students. Moreover, the study assessed these effects both immediately after the intervention and 6 months later, addressing a critical gap in educational research regarding whether the effects of the SRL intervention are sustained over time (Cleary et al., 2010, 2017; Dignath & Büttner, 2008; Wehmeyer et al., 2000). Investigating the effectiveness of SRL interventions on motivation has both theoretical and practical significance. Evidence indicates that motivational factors are among the most robust non‐cognitive predictors of students' math achievement (Živković et al., 2023), as well as key drivers of increased engagement (Galla et al., 2014; Klassen et al., 2008) and more positive emotional experiences during learning (Du et al., 2021; Li et al., 2021). Furthermore, math motivation has been identified as a crucial factor in predicting individuals' STEM career choices in the long term, often more so than affective or performance‐related factors in math (Cribbs et al., 2021; Cuder et al., 2024; Wang, 2013). In this context, testing interventions that effectively enhance motivation is essential not only for clarifying the relationship between SRL interventions and motivational outcomes, but also for providing actionable guidance to educators and policymakers seeking to implement practices that promote positive attitudes towards mathematics. This emphasis aligns with international reports (OECD, 2023) suggesting that integrating the motivational aspects into the curriculum may foster positive math achievement, in accordance with empirical evidence (Granello et al., 2025; Zakariya, 2022). This is especially important in middle school, a critical developmental period characterized by a decline in motivation and central to the formation of students' career identity (Jacobs et al., 2002; Ozturk et al., 2024). In the following sections, we provide a more detailed analysis of SRL and its interplay with the motivational factors examined in this study.

### Self‐regulated learning

SRL can be conceptualized as a complex, cyclical and dynamic process through which students regulate cognitive, affective‐motivational and behavioural aspects to achieve personal goals (Schunk & DiBenedetto, 2020; Theobald, 2021; Zimmerman & Moylan, 2009; Zimmerman & Schunk, 2011). SRL places motivation at its core: Beyond metacognitive and behavioural control, it emphasizes motivational control as the key driver of persistence, attention and goal achievement (Alonso‐Tapia & Fernandez, 2008; Panadero & Alonso‐Tapia, 2014; Paris et al., 2001). According to various theoretical frameworks (Panadero, 2017; Zimmerman & Moylan, 2009), SRL models share a common structure of interconnected phases: a planning phase, a monitoring phase and an evaluating phase. In the planning phase, students construct a representation of the task, engaging in planning strategies and goal setting. During the monitoring phase, students employ cognitive processes to tackle the task while using strategies to monitor and regulate their performance. Finally, in the evaluating phase, students assess their performance through self‐evaluation and regulation (Panadero, 2017).

Research shows that, beyond having higher academic performance (Theobald, 2021), self‐regulated learners also display higher motivation and positive attitudes in learning (Boekaerts & Corno, 2005; Efklides, 2011; Theobald, 2021; Winne, 1995; Zimmerman, 2001, 2011). For instance, studies indicate that students who demonstrate higher levels of SRL do not only achieve superior academic math performance but also exhibit enhanced motivational outcomes (Cleary et al., 2021; Cleary & Kitsantas, 2017; Ha et al., 2023; Paz‐Baruch & Hazema, 2023). However, research specifically targeting the promotion of math‐related motivational factors through SRL interventions remains scarce, especially considering middle school students (for a review, see Granello et al., 2025).

### Motivational factors and self‐regulated learning

Motivational factors play a significant role in academic achievement (Pajares & Graham, 1999; Radišić & Baucal, 2024; Schukajlow et al., 2023; Stevens et al., 2004) as well as in career interests and choices (Bargmann et al., 2022; Cuder et al., 2024; Kniveton, 2004; Murphy et al., 2019; Wang & Degol, 2013). Furthermore, these factors are central in SRL, encouraging students to initiate and sustain learning activities (Cleary & Kitsantas, 2017; Panadero, 2017; Zimmerman & Moylan, 2009). In this context, the directionality between SRL strategies and motivational outcomes remains an open and debated issue in the literature. Zimmerman and Moylan's (2009) cyclical model conceptualizes the relationship between SRL and motivational outcomes as deeply intertwined and bidirectional. Specifically, the model suggests that the use of SRL strategies can lead to enhanced motivation, which in turn fosters the continued and effective use of SRL strategies in a recursive cycle (Berger & Karabenick, 2011; Zimmerman & Moylan, 2009). However, empirical studies directly addressing the issue of directionality through cross‐lagged longitudinal designs have produced mixed findings, leaving this question unresolved and open to further investigation. Nevertheless, the effects of SRL interventions on motivational outcomes are supported by different meta‐analyses highlighting their overall effectiveness (Dignath & Büttner, 2008; Hattie et al., 1996; Theobald, 2021), although these reviews have not considered their specific effectiveness within the domain of math learning. Given the effectiveness of SRL interventions on motivational outcomes, this study focuses on promoting a set of motivational factors that have been identified as central to math learning and SRL: math perseverance, math self‐efficacy, math utility value, STEM vocational interest and theory of intelligence.

Math perseverance, which is defined as the ability to maintain sustained effort towards long‐term goals in math despite challenges or setbacks (Duckworth et al., 2007, 2016; Muenks et al., 2017; Seligman & Csikszentmihalyi, 2000), has been commonly related to SRL. Prior studies have shown that higher levels of SRL are associated with greater perseverance and effort when working on math tasks (Martin et al., 2021; Wilburne & Dause, 2016; Wolters & Hussain, 2015; Xu et al., 2023). In this regard, evidence suggests that SRL strategies of self‐monitoring and goal setting seem to be crucial for enhancing higher perseverance in math (Wilburne & Dause, 2016). Math self‐efficacy, defined as the belief in one's ability to successfully perform a specific task or behaviour (Bandura, 1997), is also associated with SRL. In this context, self‐efficacy is related to learners' willingness to initiate and persist in their efforts, influences their choice of strategies and shapes their task‐related mastery beliefs based on performance outcomes (Cleary & Kitsantas, 2017; Zimmerman & Moylan, 2009). According to Zimmerman and Moylan's cyclical model (2009), self‐efficacy not only motivates the use of SRL strategies during the initial stages of task execution, but also benefits from them, as they offer students reliable methods for approaching tasks, thereby further enhancing their self‐efficacy (Ramdass & Zimmerman, 2008). Another related motivational factor is math utility value which refers to the perceived usefulness related to math tasks and math learning in general (Eccles, 2009). Math utility value is central to SRL as it drives the initial approach to the task: When students recognize a task as useful, their motivation to engage with it and learn from it increases, leading them to employ more learning strategies and to motivate them in pursuing math activities (Harackiewicz et al., 2012; Jacobs & Eccles, 2000; Lauermann et al., 2017; Wigfield et al., 2008). Thus, similar to math self‐efficacy, this construct facilitates task initiation, but it may also be enhanced through SRL strategies, which increase students' perceived control over the learning process (Zimmerman & Moylan, 2009). This, in turn, can foster greater intrinsic motivation and engagement, ultimately increasing the value they assign to the task (Jacobs et al., 2002; Zimmerman et al., 2017). An additional motivational factor is STEM vocational interest, defined as an individual's motivation to engage with STEM‐related tasks, activities and careers (Hulleman et al., 2008; Renninger et al., 1992; Renninger & Hidi, 2011). STEM vocational interest is deeply intertwined with SRL and research shows that self‐regulated learners are better equipped to maintain sustained engagement, fostering a long‐lasting interest in STEM (Arvatz & Dori, 2024; Frazier et al., 2021). This process also interacts with and shapes students' possible selves, influencing their aspirations and future goals (Grant & Dweck, 2003; Markus & Nurius, 1986). Finally, theory of intelligence refers to implicit beliefs about the malleability of intelligence (Dweck, 2006, 2009). In this context, students with an incremental view of intelligence (vs. students with entity view of intelligence) see intelligence as improvable, which is associated to a greater effort and performance (Dweck et al., 1995; Dweck & Leggett, 1988). In this context, self‐regulated students draw on evaluative skills to prioritize effective strategies, make more adaptive causal attributions for success and failure, and emphasize effort over innate ability, thereby reinforcing the belief that intelligence is malleable (Burnette et al., 2013; Dweck, 1999, 2006, 2009; Dweck & Yeager, 2020).

In conclusion, motivational factors represent a crucial aspect of SRL, as they guide students' engagement, perseverance and mindset when approaching math tasks. By strengthening the key phases of SRL (i.e., planning, monitoring and evaluating) educators may also support motivational constructs such as math perseverance, math self‐efficacy, math utility value, STEM vocational interest and theory of intelligence. This synergistic approach fosters sustained growth in math performance, broadens career opportunities and contributes to overall students' academic well‐being (Granello et al., 2025; Pintrich et al., 2003; Steinmayr & Spinath, 2009).

### Self‐regulated learning interventions

SRL interventions typically involve multiple sessions in which professionals or educators guide students in applying cognitive, metacognitive and resource management strategies to enhance their learning and motivation (Theobald, 2021). Among these, SRL programmes based on Zimmerman and Moylan's Cyclical Model (2009) emphasize an iterative process in which learners continually regulate their thoughts, motivation and behaviours to meet personal goals. By repeatedly monitoring their progress and adjusting strategies as needed, students become more adaptive and autonomous in their learning. Multiple meta‐analyses have consistently shown that SRL interventions enhance students' academic achievement (de Boer et al., 2018; Dignath & Büttner, 2008; Theobald, 2021), while less studies have investigated the impact of SRL interventions on motivational factors (Dignath & Büttner, 2008; Hattie et al., 1996; Theobald, 2021).

In the math domain, relatively few studies have investigated how SRL interventions simultaneously affect math‐related motivational factors and math performance, highlighting the need for additional research. Preliminary evidence indicates that SRL interventions not only improve students' math performance (Granello et al., 2025; Ramdass & Zimmerman, 2008; Yıldızlı & Saban, 2016) but also foster math‐related motivational constructs such as math self‐efficacy (Falco et al., 2010; Granello et al., 2025; Ramdass & Zimmerman, 2008) and math perseverance (Wilburne & Dause, 2016). More specifically, a systematic review by Granello et al. (2025) found that SRL interventions enhance math skills while finding mixed improvements in math self‐efficacy among middle school students. With respect to math perseverance, Wilburne and Dause (2016) found that teaching students to self‐monitor and goal‐setting strategies increased their persistence in math. Complementary research has further shown that utility‐value interventions, although conceptually distinct from SRL, can enhance students' motivational beliefs and engagement (Durik et al., 2015; Hulleman & Harackiewicz, 2009), mechanisms that may indirectly support SRL processes. However, there remains a significant gap in studies adopting a comprehensive framework to examine how SRL influences multiple math‐related motivational factors at once. This gap is notable given evidence that SRL can interact with specific motivational beliefs (Boekaerts & Corno, 2005; Efklides, 2011; Winne, 1995; Zimmerman, 2001, 2011) and its enhancement positively impact both math performance and students' overall learning motivation (Dignath & Büttner, 2008). While some interventions aim to integrate SRL principles with motivational components, most are limited to assessing one or two motivational factors (Cleary et al., 2017; Dignath & Büttner, 2008; Zepeda et al., 2015). Furthermore, many SRL interventions fail to evaluate their persistent effects, particularly on motivational factors, leaving an important dimension of their long‐term efficacy unexplored (de Boer et al., 2018; Dignath & Büttner, 2008).

### The present study

Although several studies have investigated the effectiveness of SRL interventions in enhancing math performance (Dignath & Büttner, 2008; Hattie et al., 1996; Theobald, 2021), there remains a lack of research on the impact of SRL interventions on math‐related motivational factors. Additionally, there is no clear consensus on whether the effects of such interventions are sustained over time (Cazan, 2022; Cleary et al., 2017; de Boer et al., 2018; Dignath & Büttner, 2008; Lim & Yeo, 2021; Zepeda et al., 2015). Thus, the current study aimed to evaluate the effectiveness of a SRL intervention, grounded in Zimmerman and Moylan's (2009) Cyclical Model, in fostering both math‐related motivational factors and math performance among middle school students. Specifically, this study focuses on promoting a set of math‐related motivational factors that have been identified as central to math learning and SRL: math perseverance, math self‐efficacy, math utility value, STEM vocational interest and theory of intelligence. Specifically, constructs such as math self‐efficacy and math utility value were selected, as they are not only strong predictors of math learning but also core components of SRL theoretical models. These constructs are thought to both drive the use of SRL strategies during the initial stages of task engagement and benefit from such strategies by enabling more effective task management (Ramdass & Zimmerman, 2008; Zimmerman & Moylan, 2009). We also included math perseverance, as evidence indicates that SRL strategies such as self‐monitoring and goal‐setting can support students' persistence in completing math tasks (Wilburne & Dause, 2016). Additionally, we chose to examine the theory of intelligence, which, although not a math‐specific learning factor, is strongly associated with math achievement and appears to benefit particularly from SRL strategies. These strategies may help students adopt more adaptive, goal‐oriented causal attributions (Burnette et al., 2013; Dweck, 1999, 2006, 2009; Dweck & Yeager, 2020). Finally, the present study also aims to evaluate the effectiveness of an SRL intervention on students' vocational interest in STEM, given evidence that students with stronger SRL skills tend to show sustained engagement, which may ultimately lead them to pursue STEM‐related careers (Arvatz & Dori, 2024; Frazier et al., 2021).

Thus, this study had two specific aims:

1. Does the SRL intervention significantly enhance math‐related motivational factors (i.e., math perseverance, math self‐efficacy, math utility value, STEM vocational interest and theory of intelligence) and math performance, compared to a control group? Based on the current literature, we hypothesized that our SRL intervention will lead to improvements in both math‐related motivational factors and math performance at the immediate post‐test. More specifically, we expected that the intervention will have a positive effect on math perseverance given that improving students' monitoring skills supports more effective management of their resources and sustained effort towards goal attainment (Zepeda et al., 2015; Zimmerman & Moylan, 2009), as corroborated by evidence in the literature (e.g., Wilburne & Dause, 2016). We expected an enhancement in students' theory of intelligence, as the SRL intervention should help them develop better evaluative strategies and more adaptive causal attributions for success and failure (Burnette et al., 2013; Dweck, 2006, 2009). By becoming more aware that their performance can improve through instruction and practice, students should increasingly adopt an incremental rather than a fixed conception of intelligence (Dweck & Yeager, 2020). We also expected an effect of the intervention on math self‐efficacy, as developing students' SRL planning skills can provide students with practical strategies to draw upon whenever they feel uncertain about their abilities, consequently boosting their confidence (Ramdass & Zimmerman, 2008). We also expected the intervention to affect math utility value and STEM vocational interest since greater control over one's learning process tends to enhance intrinsic motivation and engagement with the task at hand (Jacobs et al., 2002; Zimmerman et al., 2017). This increased sense of control and interest can deepen students' appreciation for the value and relevance of mathematics (Wigfield & Eccles, 2002) and, ultimately, foster a stronger vocation towards STEM fields (Hidi & Renninger, 2006). Finally, we expected an effect of the SRL intervention on math performance, since several intervention studies have found a positive impact of SRL interventions on math performance at middle school level (see Dignath & Büttner, 2008 for a meta‐analysis and Granello et al., 2025 for a systematic review; Falco et al., 2010; Ramdass & Zimmerman, 2008; Yıldızlı & Saban, 2016).
2. Does the SRL intervention lead to persistent effects on math‐related motivational factors and math performance at a six‐month follow‐up? We hypothesized that our intervention would have a lasting effect on math perseverance, math self‐efficacy, math utility value, STEM vocational interest, theory of intelligence and math performance after 6 months. This hypothesis aligns with Zimmerman and Moylan's (2009) Cyclical Model, which posits that the different phases of the model mutually reinforce over time as students engage in the implementation of SRL strategies in their math learning activities.

The present study introduces some theoretical novelties. First, it examines the effects of an SRL intervention on multiple math‐related motivational factors and math performance, offering a comprehensive evaluation of how this intervention can enhance diverse math‐related outcomes. In doing so, it also considers its effects on STEM vocational interest, an underexplored motivational factor in mathematics, thereby broadening the scope of previous research. Second, the study addresses a critical gap in the literature by assessing both the immediate and persistent effects (6‐month follow‐up) of the SRL intervention on math‐related motivational factors and math performance. Given that few studies have explored these outcomes at follow‐up (de Boer et al., 2018; Dignath & Büttner, 2008), this study provides valuable insights into the intervention's persistent efficacy. Third, it specifically targets middle school students, a crucial developmental period marked by a decline in positive attitudes towards mathematics (Jacobs et al., 2002; Ozturk et al., 2024) and the early formation of professional identities that influence future STEM career choices (Cuder et al., 2024; Porfeli & Lee, 2012; Rosenzweig & Wigfield, 2016). By addressing these aspects, the findings offer meaningful implications for educators and practitioners seeking evidence‐based strategies to foster sustained academic engagement and long‐term improvements in math.

## METHOD

### Participants

A total of 412 Italian Year 6 and Year 7 middle school students were enrolled in the study. Twenty students were excluded before the start of the pretest assessment for the following reasons: *n* = 6 did not understand the Italian language; *n* = 6 presented a diagnosis of severe neuro‐developmental disorder; and *n* = 8 had a specific learning disability. Despite efforts to maximize participant retention throughout the study, seven participants were excluded from the analysis because they did not provide data at any time point (pretest, post‐test and follow‐up).

The final sample consisted of 382 middle school students (Year 6 = 189; Year 7 = 193; M_age_ = 11.37; SD_age_ = .71; Females =196; Males =186) from three different schools. Schools' classes (*n* = 21) were randomly assigned to either the intervention group (*n*_class_ = 11; *n* = 197 students; M_age_ = 11.37; SD_age_ = .75; Females = 95; Males = 102) or to the control group (*n*_class_ = 10; *n* = 185 students; M_age_ = 11.42; SD_age_ = .66; Females = 101; Males = 84). All participants were retained in the analyses (*N* = 382), including students with low intervention attendance. A small subgroup (*n* = 11) missed five or more of the six intervention sessions. Participants were middle‐class SES on average, according to their school records. All parents signed an informed consent authorizing their children's participation in the study. The study was conducted in accordance with the Declaration of Helsinki and the ethical guidelines of the Italian Association of Psychology. The study was approved by the ethical committee of the University of Trieste.

### Procedure

We employed a pretest–post‐test control group design with a 6‐month follow‐up to examine the effects of the intervention on students' math‐related motivational factors and math performance. The study consisted of four phases: the pretest assessment phase, the intervention phase, the post‐test assessment phase and the follow‐up assessment phase.

#### Pretest, post‐test and follow‐up assessment phases

The pretest, post‐test and follow‐up assessments employed the same instruments and procedures. The post‐test assessment was administered 1 week after the intervention, while the follow‐up assessment took place 6 months later. During each assessment phase, participants completed questionnaires measuring motivational factors (i.e., math perseverance, math self‐efficacy, math utility value, STEM vocational interest and theory of intelligence), along with three tasks assessing mathematical performance. All assessments were conducted collectively in the classroom and lasted approximately 60 min. The researchers conducting the students' assessments were different from those delivering the intervention and were blind to which classes belonged to the SRL intervention group.

#### Intervention phase

One week after the pretest assessment, students in the SRL intervention group began the intervention, while those in the control group attended regular math instruction covering the same mathematical content as the SRL intervention group. The intervention was delivered to the whole class by the researcher, whereas the control group activities were conducted by the teacher. Both groups participated in six weekly 60‐min sessions over a six‐week period, held between October and November 2023.

##### SRL intervention group

The intervention comprised six sessions grouped into three phases of the SRL Cyclical Model (Zimmerman & Moylan, 2009; see also Panadero, 2017), each spanning two sessions. Drawing on meta‐analytic findings of SRL interventions (Dignath & Büttner, 2008), each session was designed to foster metacognitive reflection on a specific topic and to implement strategic approaches for planning, monitoring and evaluating math performance. Specifically, the sessions were conducted as follows:

- Planning Phase (session 1 and session 2). In these sessions, students learned to interpret the characteristics of math tasks, set goals and plan strategies for solving them. First, researchers held a discussion with the students focused primarily on planning and strategies to adopt before attempting a math task. Next, students were introduced to a checklist of strategies designed to guide goal setting and plan their approach to mathematical tasks. In the end, students worked on math word problems (e.g., real‐world and abstract math word problems), algebraic expressions (e.g., combinations of terms involving addition, subtraction, multiplication and division), mental calculation (e.g., one‐digit and two‐digit additions, subtractions and multiplications) and math reasoning tasks (e.g., completion of number series). During this phase, students were presented with math tasks and asked to identify each task's features and perceived difficulty both before and after solving it, while also reflecting on any mistakes.
- Monitoring Phase (session 3 and session 4). These sessions focused on monitoring math performance, applying strategies to solve math tasks and managing errors and time effectively. First, researchers held a discussion with the students that primarily addressed how to monitor their performance while working on the math tasks. Next, students were given a checklist to support their work. The checklist, for instance, guided them in handling mistakes, seeking clarification for unclear aspects, requesting help by the peers and teacher when needed, and adhering to time constraints. In these sessions, students practiced algebraic expressions, mental calculation and arithmetic tasks (e.g., two‐digit and three‐digit additions, subtractions, multiplications and divisions).
- Evaluation Phases (session 5 and session 6). In the final sessions, students were guided to evaluate their own math performance, reflect on their errors and identify areas for improvement. First, during the discussion with the students, researchers primarily focused on how to evaluate the solving process after completing each math task and identify areas for improvement. Then, students were given a checklist to help identify weaknesses in their performance and introduce strategies to address them. They were asked to solve math tasks and then classify their mistakes (e.g., calculation errors, knowledge errors or time management errors). This process was followed by a metacognitive reflection, prompting them to recognize weaknesses in their performance and propose strategies for enhancement. The exercises in these sessions involved algebraic expressions and math reasoning tasks.

##### Control group

**FIGURE 1:**
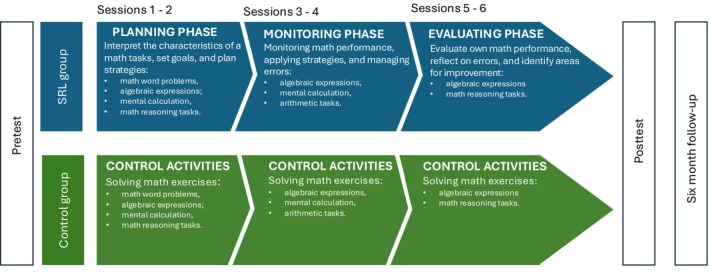
Overview of activities for each session for SRL group and control group (CG).

Students in the control group practiced on the same mathematics exercises as the SRL intervention group. Over the course of the six sessions, these activities mirrored those presented to the intervention group: In sessions 1–2, students practiced math word problems, algebraic expressions, mental calculation and math reasoning tasks; in sessions 3–4, they focused on algebraic expressions, mental calculation and arithmetic tasks; and in sessions 5–6, they continued working on algebraic expressions and math reasoning tasks. Teachers were provided with a checklist of exercises to confirm adherence. However, unlike the intervention group, students in the control group did not engage in the SRL‐specific activities (see Figure 1).

### Measures

#### Math perseverance

To assess math perseverance, we administered a questionnaire consisting of 7 items, wherein participants were asked to evaluate their perseverance in studying mathematics (adapted for mathematics from *Study Approach Questionnaire, Study Perseverance Scale* of *AMOS 8‐15*, Cornoldi et al., 2005). Responses were recorded on a Likert scale ranging from 1 (not at all) to 5 (very much). The minimum obtainable score is 7, while the maximum is 35, with higher scores indicating higher math perseverance. The scale had sufficiently good reliability in our sample (Cronbach's *α* = .62).

#### Math self‐efficacy

To measure math self‐efficacy beliefs, we administered a 10‐item questionnaire (adapted from the *Academic Self‐Efficacy Beliefs scale*; Di Giunta et al., 2013). Participants were asked to assess how capable they feel in handling specific situations related to mathematics. Participants responded using a Likert scale ranging from 1 (not at all) to 5 (very much). The minimum score that can be obtained is 10, while the maximum is 50, with higher scores indicating higher levels of math self‐efficacy. The instrument showed good reliability in our sample (Cronbach's *α* = .82).

#### Math utility value

To measure the math utility value, participants were administered a 6‐items scale (adapted from *Fennema‐Sherman Mathematics Attitude Scale*, Mulhern & Rae, 1998). The questionnaire asked participants to rate the usefulness of mathematics in their present lives and future on a Likert scale ranging from 1 to 5 (1 = not at all; 5 = very much). Three items are positively worded (e.g., ‘I study mathematics because I know how useful it is’) and three are negatively worded (e.g., ‘Taking mathematics is a waste of time’). The three negatively worded items were reverse‐scored, and the scores were summed. Possible scores ranged from 6 to 30, with higher scores indicating a greater perceived utility of mathematics. The instrument showed a good reliability in our sample (Cronbach's *α* = .82).

#### STEM vocational interest

To measure STEM vocational interest, we developed a questionnaire consisting of four items asking participants to rate how enjoyable they find pursuing a math or science‐related career on a Likert scale from 1 to 5 (1 = not at all; 5 = very much). Two items are positively worded (e.g., ‘I will enjoy doing a job related to math’) and two are negatively worded (e.g., ‘I will not enjoy doing a job related to math’). The minimum possible score is 4, and the maximum is 20, with higher score indicating higher STEM vocational interest. The instrument showed good reliability in our sample (Cronbach's *α* = .79).

#### Theory of intelligence

To assess the entity/incremental implicit theory of intelligence, the QC scale from the *AMOS* Battery (QVC; AMOS 8‐15: Questionnaire on Beliefs—Theory of Intelligence; Cornoldi et al., 2005) was administered. The questionnaire consists of a 4‐item Likert scale, from 1 (agree) to 4 (disagree), with two items representing an entity view (e.g., ‘Your intelligence is something about you that you cannot change’) and two items reflecting an incremental view of intelligence (e.g., ‘When you learn new things, you also improve your intelligence’). The entity theory items were reverse‐scored, and an average intelligence theory score was calculated for the four items, with higher scores representing an incremental view of the intelligence. The minimum possible score is 4, and the maximum is 16. The scale had sufficiently good reliability in our sample (Cronbach's *α* = .65).

#### Math performance

To assess mathematical performance, three subtests from the Italian standardized battery for the Evaluation of calculation skills (*AC‐MT‐3*; Cornoldi et al., 2020) were administered: Calculation Fluency, Logical Inferences and Matrices. In the Calculation Fluency task, participants were presented with three lists of operations: one containing 20 addition problems, one with 20 subtraction problems, and one with 20 multiplication problems, all arranged in a column. They had 1 min to solve each list. The Logical Inference task comprised three types of problems. In the symbol‐based operations task, participants were shown equations in which digits and results were replaced by symbols, and they had to infer the value of each symbol. In the missing‐sign operations task, they were presented with equations where the arithmetic sign was missing, and they had to determine the correct sign based on the provided result. In the incomplete operations task, they were given two equations, one of which lacked a result, and were required to find the solution to the incomplete equation without performing any calculations, relying instead on the other equation as a reference. Finally, the Matrices subtest assessed children's arithmetic and reasoning skills by asking them to identify a rule within a number series in order to determine the correct number for an empty cell. Participants had 2 min to solve 12 incomplete numerical matrices by applying the identified rule. One point was awarded for each correct response, and the points were subsequently summed. Possible scores ranged from 0 to 84, with higher scores indicating better mathematical performance. According to the instrument manual, the tasks demonstrate good reliability across the considered age groups: calculation fluency (Cronbach's *α*: Year 6 = .88; Year 7 = .92), inferences (Cronbach's *α*: Year 6 = .71; Year 7 = .67), numerical matrices (Cronbach's *α*: Year 6 = .81; Year 7 = .83).

### Analytical Strategy

All analyses were conducted in Mplus (Version 8; Muthén & Muthén, 2018). Given the clustered design (students nested within classes), intraclass correlations (ICCs) were computed for each outcome at post‐test and follow‐up (Bliese, 2000). Significant ICCs emerged for math performance (ICC_post_ = .17, *p* = .011; ICC_follow‐up_ = .17, *p* = .005), theory of intelligence (ICC_post_ = .19, *p* < .001; ICC_follow‐up_ = .15, p < .001) and math perseverance (ICC_post_ = .08, *p* = .029). All other ICCs were negligible (range = .05–.10; see Table S1).

To examine pretest differences and intervention effects at post‐test and follow‐up, we estimated multilevel models with classroom‐level random intercepts to account for the clustered structure of the data and classroom‐level randomization. To test for baseline differences, each pretest outcome was regressed on intervention group with classroom specified as a random intercept. Separate multilevel models were then estimated for each post‐test and follow‐up outcome, including intervention group, the corresponding pretest score and gender as fixed effects, and classroom as a random intercept. Pretest scores were included to control for baseline performance, and gender was added as a covariate given prior evidence that gender differences shape students' math motivational trajectories in middle school (Bohrnstedt et al., 2024; Diseth et al., 2014; Eccles & Wang, 2016; Else‐Quest et al., 2010; Meece et al., 2006). This decision is further supported by OECD data showing that Italy has the largest gender gap in math performance among member countries (OECD, 2023).

Missing data were handled with multiple imputation (*n*_dataset_ = 20). Prior to imputation, missing data patterns were examined and Little's test indicated data were not MCAR (χ^2^(317) = 481.88, *p* < .001). Imputation followed Graham's (2009) procedure using included variables as auxiliaries. We adopted an intention‐to‐treat (ITT) approach, including all randomized participants. Per‐protocol analyses (PPA; excluding students who missed ≥5 of 6 sessions, *n* = 11) are reported in the Data S1 and yielded consistent results with ITT.

Prior to the study, power analysis indicated that 210 participants were sufficient to detect a medium effect size in ANCOVA (*f* ^2^ = .15; *α* = .05; power = .95; Numerator df = 1; Number of groups = 2; Number of covariates = 2). Because ICCs suggested classroom clustering was not negligible, we adopted a multilevel approach changing our original analytical plan. A post‐hoc sensitivity analysis showed the minimum detectable effect size (MDES) for a two‐level design was .566 (95% CI [.165, .968]), indicating adequate power to detect moderate or larger effects. Full multilevel results are presented in the manuscript, with single‐level analyses provided in the Data S1 for transparency. The two analytical approaches yielded consistent results (see Data S1). Cohen's criteria (Cohen, 1988) were used to interpret effect sizes: small (*d* = .20), medium (*d* = .50) and large (*d* = .80).

## RESULTS

Descriptive statistics for each group at each time point are presented in Table 1.

**TABLE 1:** Descriptive statistics for each group at pretest, post‐test and follow‐up (Intention‐To‐Treat sample; see Data S1 for Per‐Protocol Analyses).

	Pretest		Post‐test		Follow‐up	
	SRL	CG	SRL	CG	SRL	CG
Math perseverance						
M	23.73	24.05	24.43	23.56	24.21	23.62
SD	4.52	4.43	4.05	4.35	4.04	4.38
Math self‐efficacy						
M	35.27	34.99	35.27	34.81	35.76	34.75
SD	6.53	6.32	6.31	6.04	6.57	6.55
Math utility value						
M	24.70	24.78	25.34	24.50	25.04	24.52
SD	4.15	4.04	4.09	4.50	4.20	4.29
STEM vocational interest						
M	11.93	12.58	12.42	12.37	11.93	12.50
SD	4.06	3.97	4.08	4.30	4.08	3.92
Theory of intelligence						
M	11.98	11.66	14.00	11.87	13.97	12.14
SD	2.73	2.62	1.84	2.51	2.05	2.67
Math performance						
M	35.52	32.75	41.47	35.27	41.28	34.76
SD	10.79	11.18	11.51	12.26	11.71	11.62

Abbreviations: CG, control group; M, mean; SD, standard deviation; SRL, self‐regulated learning intervention group.

Multilevel models testing baseline differences at pretest between the SRL intervention and control groups revealed no statistically significant group differences for math self‐efficacy (*B* = .312, *p* = .633), math anxiety (*B* = −.336, *p* = .461), math utility value (*B* = .335, *p* = .234), STEM vocational interest (*B* = −.235, *p* = .589) and theory of intelligence (*B* = −.647, *p* = .121). However, a statistically significant difference emerged for math performance (*B* = 2.836, *p* = .014), with higher scores in the SRL intervention group compared to the control group at baseline.

### Post‐test assessment

To evaluate post‐test differences between the SRL intervention group and the control group, a series of multilevel models were estimated for each outcome measure, controlling for the respective pretest score and gender (see Table 2). Results showed that the SRL intervention group reported statistically significantly higher scores at post‐test compared to the control group on math perseverance (*B* = 1.134, *p* = .024, *d* = .349), theory of intelligence (*B* = 2.074, *p* < .001, *d* = .973), math utility value (*B* = .998, *p* = .014, *d* = .298) and math performance (*B* = 3.184, *p* < .001, *d* = .595). No statistically significant differences were found at post‐test on math self‐efficacy (*B* = .173, *p* = .382, *d* = .038) or STEM vocational interest (*B* = .474, *p* = .259, *d* = .157).

**TABLE 2:** Multilevel regression models with group (intervention vs. control) predicting each outcome at post‐test and follow‐up level (Intention‐To‐Treat sample; see Data S1 for Per‐Protocol Analyses).

	Post‐test				Follow‐up			
	*B*	SE	*p*	*d*	*B*	SE	*p*	*d*
Math perseverance	1.134	.503	.024*	.349	.794	.404	.041*	.252
Math self‐efficacy	.173	.454	.382	.038	.695	.462	.132	.164
Math utility value	.998	.405	.014*	.298	.664	.404	.100	.205
STEM vocational interest	.474	.420	.259	.157	−.122	.366	.803	−.043
Theory of intelligence	2.074	.275	<.001***	.973	1.726	.225	<.001***	.833
Math performance	3.184	.867	<.001***	.595	4.046	1.105	<.001***	.706

Abbreviations: *B*, regression coefficient; *d*, Cohen's *d*; *p*, *p*‐value; SE, standard error.

* *p* < .05.

*** *p* < .001.

### Follow‐up assessment

To evaluate follow‐up differences between the SRL intervention group and the control group, a series of multilevel models were estimated for each outcome measure, controlling for the respective pretest score and gender (see Table 2). The results indicated that the SRL intervention group reported statistically significantly higher scores at follow‐up compared to the control group on math perseverance (*B* = .794, *p* = .041, *d* = .252), theory of intelligence (*B* = 1.726, *p* < .001, *d* = .833) and math performance (*B* = 4.046, *p* < .001, *d* = .706). No statistically significant differences were found at follow‐up between groups for math self‐efficacy (*B* = .695, *p* = .132, *d* = .164), math utility value (*B* = .664, *p* = .100, *d* = .205) or STEM vocational interest (*B* = −.122, *p* = .803, *d* = −.043).

## DISCUSSION

The general aim of the present study was to evaluate the effectiveness of an SRL intervention in fostering both math‐related motivational factors (i.e., math perseverance, math self‐efficacy, math utility value, STEM vocational interest and theory of intelligence) and math performance among middle school students. Although a large body of research has documented the positive impact of SRL interventions on academic performance (Dignath & Büttner, 2008; Hattie et al., 1996; Theobald, 2021), considerably less attention has been paid to their influence on math‐related motivational factors, especially among middle school students. By investigating this gap, the present study sought to determine whether an SRL intervention could strengthen students' math‐related motivational factors and math performance, thereby providing insights into the potential for SRL interventions to provide sustained educational benefits during middle school.

The first aim of this study was to examine the effectiveness of an SRL intervention in improving math‐related motivational factors and math performance at the immediate post‐test. Our findings indicated that the intervention significantly enhanced students' math perseverance, math utility value, theory of intelligence and math performance, but not math self‐efficacy and STEM vocational interest. These results align with prior research suggesting that SRL interventions can strengthen math perseverance (Wilburne & Dause, 2016), math utility value (Durik et al., 2015) and theory of intelligence (Burnette et al., 2023). Moreover, they align with systematic reviews indicating the efficacy of SRL interventions in promoting general academic achievement (Dignath & Büttner, 2008) and, more specifically, mathematics performance (Granello et al., 2025). A particular novelty of our study is to test the effectiveness of the SRL intervention on STEM vocational interest, given that, although motivation for STEM learning is generally influenced by SRL skills (Lei, 2024), there is limited evidence on whether SRL interventions directly enhance students' STEM vocational interest (Cuder et al., 2024; Daker et al., 2021). Following the SRL intervention, no significant effect emerged for STEM vocational interest at either post‐test or follow‐up. One possible explanation is that vocational interest represents a more stable trait that may require more intensive or personally relevant interventions to change, particularly in early adolescence. Additionally, interest in STEM careers may be shaped by broader social and cultural factors, making difficult to assess its change in the scope of a short‐term classroom‐based programme.

Notably, the intervention did not yield a statistically significant effect on math self‐efficacy, contrary to our initial hypotheses. This finding contributes to the mixed results in the literature regarding the impact of SRL interventions on math self‐efficacy (for a review, see Granello et al., 2025). One possible explanation lies in how math self‐efficacy is conceptualized: it reflects perceived competence in very specific math tasks and skills that our intervention did not directly address. In this regard, it could be speculated that the SRL intervention may have been more effective at strengthening a broader sense of math self‐competence, which aligns more closely with the math self‐concept construct (Marsh et al., 2019). An alternative explanation could be that math self‐efficacy beliefs might be relatively stable over time and largely shaped by mastery experiences (Bandura, 1997; Li et al., 2021; Usher & Pajares, 2009), rather than by the metacognitive reflection and strategy use typically addressed by SRL interventions. Future studies should include measures that better capture students' overall sense of math competence (i.e., math self‐concept), or implement instructional training that directly enhances math self‐efficacy through targeted exercises, which may prove more effective than indirectly fostering it via SRL interventions.

Our second aim was to assess the effectiveness of the SRL intervention in enhancing math‐related motivational factors and math performance six months after the intervention. The results showed that the positive effects of the SRL intervention remained statistically significant at follow‐up for math perseverance, theory of intelligence and math performance, but not for math self‐efficacy, math utility value and STEM vocational interest. This finding is particularly noteworthy because, to the best of our knowledge, no previous study has examined the persistent effects of SRL interventions on math‐related motivational factors. The obtained results support earlier evidence (de Boer et al., 2018) indicating that SRL interventions can sustain math performance gains over time. Contrary to our starting hypotheses, we observed no statistically significant difference between the groups in math utility value or STEM vocational interest at follow‐up. For math utility value, this outcome may reflect a natural decline in the intervention's effects over time, especially in the absence of additional sessions between the post‐test and follow‐up assessments. Therefore, increasing the number of intervention sessions might help sustain the intervention's effects on math utility value over time. This decline may also reflect a shift in curricular focus later in the school year towards more abstract content (e.g., algebra), which may have reduced students perceived relevance of mathematics despite the intervention's attempts. Future studies should consider tracking curricular content to better capture how classroom curricular instruction moderates the intervention's effects.

Overall, these findings highlight the potential of SRL‐based interventions to foster lasting improvements in math‐related motivation and math performance. Specifically, emphasizing the planning, monitoring and evaluation phases of the SRL Cyclical Model (Zimmerman & Moylan, 2009) may have initiated an iterative process in which learners continually regulate their thoughts, motivation, and behaviours, monitor their progress, and adjust their strategies as needed. This process likely promotes greater autonomy and adaptability in learning, ultimately leading to stronger math‐related motivation and math performance. The fact that some improvements persisted six months after the intervention suggests that these approaches can effectively support math‐related motivation and math performance over time, fostering a cyclical positive feedback loop. However, these findings also underscore the importance of ongoing support and curricular alignment of SRL interventions to maintain improvements in other motivational constructs where no persistent enhancement was observed.

### Limitations and areas of future research

The current study has some limitations that future research could address. First, our intervention consisted of only six sessions spread over a month and a half. Although this scheduling is commonly used in SRL intervention studies (Granello et al., 2025), a more intensive or extended programme might yield broader and more persistent effects on math‐related motivational factors and math performance, particularly on math self‐efficacy and STEM vocational interest. Relatedly, although we evaluated persistent intervention effects at six months, future studies might consider additional sessions between the post‐test and follow‐up or adopting multiple follow‐up assessments over a longer period to capture more nuanced trajectories of improvement. Second, although our study employed a robust set of measures to assess math‐related motivational factors, future research should more thoroughly investigate how SRL interventions can enhance math self‐efficacy. Indeed, because math self‐efficacy reflects perceived competence in specific mathematical activities, expanding the range of items assessing these activities or incorporating broader constructs of perceived competence (e.g., math self‐concept) could more clearly reveal the effects of SRL interventions on these constructs. Similarly, studies could incorporate more ecological measures (e.g., future STEM course enrolment or actual math grades) and include emotional factors (e.g., math anxiety and math enjoyment) that are equally critical in math education (Živković et al., 2023). In addition, the present study did not include a measure of students' SRL strategies, nor did it directly assess whether the intervention effectively improved students' use of SRL strategies in math. This limitation, commonly found in math SRL intervention studies (e.g., Falco et al., 2010; Ramdass & Zimmerman, 2008), represents an important area for future research. Indeed, incorporating a self‐report measure of SRL strategy use could provide a more nuanced understanding of the mechanisms through which the intervention may promote improvements in math‐related motivational factors and math performance. A further limitation concerns the delivery format of the intervention. Whereas the intervention was implemented by trained researchers, the control group was taught by regular classroom teachers. This difference may introduce potential instructor effects or expectancy biases. Moreover, prior work indicates that researcher‐delivered interventions often yield larger effects than teacher‐delivered ones (Dignath & Büttner, 2008). Although our decision ensured consistency and fidelity in the implementation, the positive outcomes may partly reflect differences in delivery format, which future research should examine more systematically, for instance through teacher‐delivered formats. Finally, replicating this intervention with more diverse samples or in different educational contexts could help assess the generalizability of our findings and shed light on how varying curricular demands, particularly those that might influence students' perceptions of math utility or interest, can affect the outcomes of SRL interventions.

## CONCLUSIONS

This study represents an initial attempt to assess both the immediate and persistent effects of an SRL intervention on middle school students' math‐related motivational factors and math performance. The findings indicate that SRL interventions can be effectively implemented to enhance both math‐related motivation and math performance, with benefits persisting even 6 months after the intervention. From a theoretical standpoint, these results offer a novel contribution to the literature, given that the impact of SRL interventions on math‐related motivational factors has been relatively underexplored (Granello et al., 2025), especially regarding their persistent effects (de Boer et al., 2018). Furthermore, this study advances prior research by incorporating a measure of STEM vocational interest, suggesting that SRL interventions may not foster interest in STEM careers. In terms of practical implications, the study underscores relevant considerations for educators and professionals. Notably, the intervention was delivered in a classroom setting, which underscores its adaptability and ecological validity for groups of students rather than individuals. In this context, even a brief SRL programme can lead to improvements not only in math performance, as documented in previous research (Dignath & Büttner, 2008; Granello et al., 2025), but also in math‐related motivational factors, which seem to be maintained over time. Consequently, SRL interventions can equip students with strategies that enhance learning effectiveness while also helping them manage the negative attitudes that often accompany the math learning process, thereby making math learning a more accessible experience for all students.

## AUTHOR CONTRIBUTIONS

**Federica Granello:** Conceptualization; investigation; methodology; data curation; writing – original draft. **Alessandro Cuder:** Writing – review and editing; formal analysis; methodology. **Eleonora Doz:** Conceptualization; writing – review and editing; methodology. **Sandra Pellizzoni:** Writing – review and editing; conceptualization; supervision. **Maria Chiara Passolunghi:** Supervision; data curation; methodology; conceptualization; writing – review and editing.

## CONFLICT OF INTEREST STATEMENT

None of the authors have a conflict of interest to disclose.

## Supporting information


Data S1.

## Data Availability

The data that support the findings of this study are available on request from the corresponding author. The data are not publicly available due to privacy or ethical restrictions.
