# Supplementary material for: “I can do math!”: A self‐regulated learning intervention to enhance math‐related motivational factors and performance in middle school
Source: Br J Educ Psychol. 2025 Sep 18;96(2):501–21. doi: 10.1111/bjep.70034 (PMC13155042; doi:10.1111/bjep.70034)
Supplement: Supplementary file 1 — Data S1. [file BJEP-96-501-s001.docx]

**Supplementary Materials**

**Supplementary Table 1**

Intraclass correlation coefficents (ICCs) for each outcome.

|  | **Posttest** | | | **Follow-up** | | |
| --- | --- | --- | --- | --- | --- | --- |
|  | **ICC** | ***F*** | ***p*** | **ICC** | ***F*** | ***p*** |
| Math perseverance | 0.078 | 0.075 | .029^*^ | 0.044 | 0.020 | .324 |
| Math self-efficacy | 0.014 | 0.002 | .957 | 0.021 | 0.008 | .722 |
| Math utility value | 0.049 | 0.037 | .159 | 0.033 | 0.031 | .287 |
| STEM vocational interest | 0.035 | 0.014 | .516 | 0.031 | 0.016 | .812 |
| Theory of intelligence | 0.188 | 0.250 | <.001^***^ | 0.149 | 0.120 | <.001^***^ |
| Math performance | 0.170 | 1.83 | .011^*^ | 0.168 | 0.180 | .005^**^ |

*Note*. ICC = intra-class correlation coefficient; *F* = *F* statistic, *p* = *p*-value; *^*^p* < .05, ^*^*^*^p* < .01, ^***^*p* < .001

**Supplementary Table 2**

Per-Protocol Analysis (PPA): descriptive Statistics for each group at pretest, post-test and follow-up.

|  |  | **Pretest** | | **Posttest** | | | **Follow-up** | | |
| --- | --- | --- | --- | --- | --- | --- | --- | --- | --- |
|  |  | **SRL** | **CG** | **SRL** | **CG** | **SRL** | | **CG** |  |
| Math perseverance | M | 23.81 | 24.02 | 24.47 | 23.52 | 24.30 | | 23.60 |  |
|  | SD | 4.28 | 4.42 | 4.21 | 4.34 | 4.02 | | 4.38 |  |
| Math self-efficacy | M | 35.34 | 34.88 | 35.36 | 34.77 | 35.81 | | 34.73 |  |
|  | SD | 6.33 | 6.32 | 6.65 | 6.03 | 6.51 | | 6.56 |  |
| Math utility value | M | 24.69 | 24.90 | 25.37 | 24.51 | 25.00 | | 24.50 |  |
|  | SD | 4.29 | 4.05 | 4.13 | 4.50 | 4.22 | | 4.30 |  |
| STEM vocational interest | M | 12.10 | 12.61 | 12.63 | 12.39 | 12.06 | | 12.53 |  |
|  | SD | 4.00 | 3.96 | 4.04 | 4.30 | 4.06 | | 3.91 |  |
| Theory of intelligence | M | 11.97 | 11.66 | 13.99 | 11.86 | 13.96 | | 12.13 |  |
|  | SD | 2.62 | 2.63 | 1.96 | 2.51 | 2.04 | | 2.68 |  |
| Math performance | M | 35.37 | 32.76 | 41.31 | 35.75 | 41.22 | | 34.80 |  |
|  | SD | 10.74 | 11.21 | 11.31 | 12.29 | 11.31 | | 11.65 |  |

*Note.* M = Mean; SD = Standard Deviation; SRL = Self-regulated learning intervention group; CG = control group.

**Supplementary Table 3**

Per-Protocol (PP): Multilevel Models with group (intervention vs control) predicting each outcome at post-test and follow-up level, controlling for gender and the outcome at the pretest, with classroom-level random intercepts.

|  | **Posttest** | | | | **Follow-up** | | | |
| --- | --- | --- | --- | --- | --- | --- | --- | --- |
|  | ***B*** | **S.E.** | ***p*** | ***d*** | ***B*** | **S.E.** | ***p*** | ***d*** |
| Math perseverance | 1.142 | 0.502 | .023^*^ | 0.351 | 0.832 | 0.394 | .035^*^ | 0.264 |
| Math self-efficacy | 0.255 | 0.461 | .553 | 0.056 | 0.755 | 0.423 | .074 | 0.177 |
| Math utility value | 1.007 | 0.414 | .015^*^ | 0.298 | 0.640 | 0.419 | .127 | 0.196 |
| STEM vocational interest | 0.539 | 0.431 | .211 | 0.178 | -0.121 | 0.385 | .753 | -0.043 |
| Theory of intelligence | 2.073 | 0.278 | <.001^***^ | 0.975 | 1.774 | 0.212 | <.001^***^ | 0.855 |
| Math performance | 3.188 | 0.889 | <.001^***^ | 0.591 | 4.088 | 1.12 | <.001^***^ | 0.711 |

*Note*. *B* = regression coefficient, S.E.= standard error, *p* = *p*-value; *d* = Cohen’s *d*.
*^*^p* < .05, ^**^ p < .01, ^***^*p* < .001

**Supplementary Table 4a**

Intention-To-Treat (ITT): Linear Models with group (intervention vs control) predicting each outcome at post-test and follow-up level, controlling for gender and the outcome at the pretest.

|  | **Posttest** | | | | **Follow-up** | | | |
| --- | --- | --- | --- | --- | --- | --- | --- | --- |
|  | ***B*** | **S.E.** | ***p*** | ***d*** | ***B*** | **S.E.** | ***p*** | ***d*** |
| Math perseverance | 1.060 | 0.372 | .004^**^ | 0.315 | 0.776 | 0.394 | .044^*^ | 0.178 |
| Math self-efficacy | 0.197 | 0.480 | .682 | 0.043 | 0.722 | 0.472 | .127 | 0.110 |
| Math utility value | 0.976 | 0.375 | .009^**^ | 0.287 | 0.640 | 0.389 | .100 | 0.149 |
| STEM vocational interest | 0.479 | 0.331 | .148 | 0.155 | -0.142 | 0.339 | .675 | -0.036 |
| Theory of intelligence | 2.060 | 0.232 | <.001^***^ | 0.954 | 1.694 | 0.231 | <.001^***^ | 0.634 |
| Math performance | 3.165 | 0.622 | <.001^***^ | 0.572 | 4.083 | 0.743 | <.001^***^ | 0.351 |

*Note*. *B* = regression coefficient, S.E.= standard error, *p* = *p*-value; *d* = Cohen’s *d*.
*^*^p* < .05, ^**^ p < .01, ^***^*p* < .001

**Supplementary Table 4b**

Per-Protocol Analysis (PP): Linear Models with group (intervention vs control) predicting each outcome at post-test and follow-up level, controlling for gender and the outcome at the pretest.

|  | **Posttest** | | | | **Follow-up** | | | |
| --- | --- | --- | --- | --- | --- | --- | --- | --- |
|  | ***B*** | **S.E.** | ***p*** | ***d*** | ***B*** | **S.E.** | ***p*** | ***d*** |
| Math perseverance | 1.074 | 0.378 | .004^**^ | 0.254 | 0.823 | 0.395 | .037^*^ | 0.188 |
| Math self-efficacy | 0.270 | 0.487 | .580 | 0.043 | 0.717 | 0.477 | .133 | 0.109 |
| Math utility value | 0.990 | 0.382 | .010^**^ | 0.241 | 0.627 | 0.401 | .118 | 0.146 |
| STEM vocational interest | 0.573 | 0.339 | .091 | 0.139 | -0.126 | 0.355 | .723 | -0.032 |
| Theory of intelligence | 2.063 | 0.235 | <.001^***^ | 1.037 | 1.697 | 0.237 | <.001^***^ | 0.636 |
| Math performance | 3.175 | 0.636 | <.001^***^ | 0.272 | 4.138 | 0.748 | <.001^***^ | 0.356 |

*Note*. *B* = regression coefficient, S.E.= standard error, *p* = *p*-value; *d* = Cohen’s *d*.
*^*^p* < .05, ^**^ p < .01, ^***^*p* < .001

**Measures**

**Math self-efficacy** adapted from the *Academic Self-Efficacy Beliefs scale*; Di Giunta et al., 2013

Skills sub-scale

How well…

1. …do you learn mathematics?
2. …do you solve math problems?
3. …do you do mental calculations?
4. …do you solve written operations?
5. …do you recall multiplication tables?

Organizational sub-scale

How well…

1. …do you finish your assigned math homework?
2. …do you stay focused while studying math without getting distracted?
3. …do you study math even when there are other interesting things to do?
4. …do you organize your school activities?
5. …do you take interest in school subjects?

**Math perseverance** adapted for mathematics from *Study Approach Questionnaire, Study Perseverance Scale* of *AMOS 8-15*, Cornoldi et al., 2005.

1. If I can’t solve a math exercise, I keep trying until I understand where I went wrong.
2. When the teacher assigns math homework, I only work on the problems that interest me.
3. I enjoy studying math to learn new things.
4. If I have a lot of math to study, I give up doing other things I like.
5. In math, I study only the bare minimum to get a passing grade.
6. I don’t like leaving a math-related study activity unfinished.
7. Even if I haven’t fully understood a math topic, I still try to complete the assigned exercises.

**Math utility** adapted from *Fennema-Sherman Mathematics Attitude Scale*, Mulhern & Rae, 1998.

1. I study mathematics because I know how useful it is.
2. Knowing mathematics will help earn a living.
3. Mathematics is a worthwhile and necessary subject.
4. Mathematics will not be important to me in my life's work.
5. Mathematics is of no relevance for my life.
6. Taking mathematics is a waste of time.

**Theory of Intelligence** from the *AMOS* Battery *QVC; AMOS 8-15: Questionnaire on Beliefs*, Cornoldi et al., 2005.

1. Your intelligence is something about you that you cannot change.
2. You can learn new things, but you cannot change your intelligence.
3. No matter how much intelligence you have, you can always change it at least a little.
4. When you learn new things, you also improve your intelligence.

**STEM vocational interest**

1. I will enjoy doing a job related to math.
2. I will not enjoy doing a job related to science.
3. I will enjoy doing a job related to math.
4. I will not enjoy doing a job related to science.
